# RCSB Protein Data Bank 1D tools and services

**DOI:** 10.1093/bioinformatics/btaa1012

**Published:** 2020-12-27

**Authors:** Joan Segura, Yana Rose, John Westbrook, Stephen K Burley, Jose M Duarte

**Affiliations:** btaa1012-aff1 Research Collaboratory for Structural Bioinformatics Protein Data Bank, San Diego Supercomputer Center, University of California, La Jolla, CA 92093, USA; btaa1012-aff2 Research Collaboratory for Structural Bioinformatics Protein Data Bank, Rutgers, The State University of New Jersey, Piscataway, NJ 08854, USA; btaa1012-aff3 Institute for Quantitative Biomedicine, Rutgers, The State University of New Jersey, Piscataway, NJ 08854, USA; btaa1012-aff4 Department of Chemistry and Chemical Biology, Rutgers, The State University of New Jersey, Piscataway, NJ 08854, USA; btaa1012-aff5 Cancer Institute of New Jersey, Rutgers, The State University of New Jersey, New Brunswick, NJ 08901, USA

## Abstract

**Motivation:**

Interoperability between polymer sequences and structural data is essential for providing a complete picture of protein and gene features and helping to understand biomolecular function.

**Results:**

Herein, we present two resources designed to improve interoperability between the RCSB Protein Data Bank, the NCBI and the UniProtKB data resources and visualize integrated data therefrom. The underlying tools provide a flexible means of mapping between the different coordinate spaces and an interactive tool allows convenient visualization of the 1-dimensional data over the web.

**Availabilityand implementation:**

https://1d-coordinates.rcsb.org and https://rcsb.github.io/rcsb-saguaro.

**Supplementary information:**

Supplementary data are available at *Bioinformatics* online.

## 1 Introduction

With rapid improvements in genome sequencing technologies and protein structure determination tools, bioinformatic resources compiling gene and protein structure information are growing faster than ever and new biochemical and molecular content is released weekly if not daily. Currently, the NCBI RefSeq database contains >178 million non-redundant protein sequences and >32 million transcripts (Sharma et al., 2019). To date, UniProtKB has manually annotated >500 000 proteins, and >120 million proteins have been computationally annotated (UniProt, 2019). More than 166 000 three-dimensional (3D) structures of biomolecules (proteins, DNA and RNA) have been deposited into the Protein Data Bank (PDB), the single global archive of experimental macromolecular structure data. In the USA, these data are made available through the RCSB PDB web portal (Burley *et al.*, 2019). Maintaining residue-level correspondences across the spectrum of data hosted by these three resources has become a complex yet essential task for providing complete and consistent views of the biological features of genes and proteins. Combing these alignments with an integrated visual presentation is required to enable users to understand how 3D structures and structural changes influence biochemical properties and biological function.

The problem of providing protein-to-genome sequence alignments is not fully solved at present. For example, the Structure Integration with Function, Taxonomy and Sequence (SIFTS) uses a semiautomatic pipeline to compute residue level mappings between sequences in PDB 3D structures and proteins in UniProtKB (Dana et al., 2019). The UniProtKB resource provides protein sequence alignments with ENSEMBL (Zerbino et al., 2018) genome data. Other services provide genome to 3D protein structure alignments for Human data (Solomon et al., 2016; Wang et al., 2018). While these resources have implemented web application programming interfaces (APIs) for access, there are no general services that generate residue alignments between protein structures and genome sequences for all organisms present in the structural archive. Moreover, mapping positional annotations among databases is not provided by any of these resources.

In addition to structure–protein–gene sequence alignment integration, facile data visualization is critical for understanding gene and protein biological features. Graphical summaries of sequence annotations enable identification of relationships and patterns among positional features. Most existing graphical libraries that display positional features were originally developed around individual data resources (Jaschob et al., 2015; Mukhyala and Masselot, 2014; Watkins *et al.*, 2017). Recently, the Feature-Viewer (Paladin et al., 2020) and Nightingale (https://github.com/ebi-webcomponents/nightingale) were designed as general tools to display annotations over sequences; however, these tools and other extant libraries were principally designed to display biological features for a single sequence. As such, they are not well suited for visualization of positional annotations integrated from multiple sources, a key requirement of the current structure–protein–gene integration framework where one-to-many relationships commonly occur among databases.

Herein, we present two new RCSB PDB resources: the 1D Coordinate Server and the 1D Protein Feature Viewer. Both tools were developed to simplify data integration and visualization of gene and protein features at both sequence and 3D structural levels. Moreover, these tools have significantly broadened data integration of RCSB PDB structural data with gene and protein feature annotations available from NCBI and UniProtKB. The 1D Coordinate Server is a web service that maintains a weekly updated mapping among NCBI, UniProtKB and PDB structure data. This resource provides both relationships between database entries and alignments of protein sequences and alignment of protein sequences with genome sequences. The second resource, the 1D Protein Feature Viewer, is a new library developed to display sequence positional features using a web browser client. The motivation for this development was to provide a collection of modern performant tools capable of displaying structural and biological features integrated from multiple sequence references in a context of one-to-many relationships and alignments containing gaps.

## 2 Tools

**1D Coordinate Server:** The 1D Coordinate Server is a web service that integrates information at protein and genome level from NCBI, UniProtKB and RCSB PDB. Integrated data includes both residue level mappings between protein and genome sequences drawn the different databases and positional annotations collected from UniProtKB and PDB structures. Alignments are encoded into JSON format documents that contain aligned regions as arrays of ranges (see Supplementary Sections S1 and S2). Protein sequence to genome sequence alignments are organized such that each residue is mapped to three consecutive nucleotides starting from the initial position. In some situations, protein residues may mismatch with the exon terminal nucleotides, in these cases shifted nucleotides belonging to the subsequent exon are encoded in a separate array. In addition, protein to genome sequence alignments include DNA strand orientation to indicate the direction in which alignments should be assembled. Integration of protein positional annotation includes the full range of biological features available in UniProtKB (e.g. post-translational modifications, genome variants, linear motifs) and 3D features extracted from PDB structures (e.g. secondary structure, ligand binding sites).

The server combines two independent architectural components. First, a data pipeline collects and integrates genome and protein information from NCBI and UniProtKB with RCSB PDB structural data. Second, a Java based web service efficiently loads and dispatches the integrated data through a GraphQL web API. Relationships between NCBI RefSeq and UniProtKB entries are obtained from the Protein Information Resource (Wu, 2003), and then mapped to PDB structures through their associated UniProtKB sequences. Thereafter, the data pipeline collects alignments between UniProtKB and PDB from SIFTS. Alignments from UniProtKB to NCBI RefSeq and PDB sequences are computed using the Smith-Waterman algorithm as implemented in BioJava (Lafita et al., 2019). Residue mappings from protein sequence to genome sequence are completed through NCBI data, using alignments of RefSeq proteins to their reference genomes. This information is in turn used to bridge UniProtKB and PDB sequences with NCBI reference genomes (for a detailed description of how alignments between the different sequence databases are obtained see Supplementary Section S3). Finally, alignments are stored in a document-oriented database (MongoDB) that serves as the data source for the web service. The underlying computational pipeline is executed weekly to maintain up-to-date mappings at residue/nucleotide levels among NCBI, UniProtKB and PDB.

The server supports two request types: first, alignments between protein sequences and alignments between protein sequences and genome sequences, and second, integrated annotations from UniProtKB and structural features from RCSB PDB mapped onto any integrated reference.

*Alignment requests:* The server delivers alignments between any of the integrated databases, including alignments between protein sequences and genome sequences. For example, the genome coordinates for all PDB structures that map to a given chromosome or the alignments between a given NCBI entry and all its related PDB structures.

*Annotation requests:* UniProtKB and RCSB PDB positional annotations can be mapped onto any of the integrated databases including genome sequences and protein sequences. For example, all protein-ligand binding sites annotated in PDB structures can be mapped at the chromosome level. Post-translational modifications of a particular UniProtKB sequence can also be mapped over PDB structures or NCBI RefSeq entries.

**1D Protein Feature Viewer:** The 1D Protein Feature Viewer is an open-source library (https://github.com/rcsb/rcsb-saguaro) written in TypeScript designed for visualization of biological features of proteins on the web (Supplementary Fig. S2). The library provides core tools for displaying positional annotations over linear domains using multiple type of representations (block, pin, bond, curves, etc.). The primary motivation behind this development was to display annotations when multiple sequence references are available. For example, common occurrences in PDB structures are deletion or insertion of protein regions or chimeric macromolecules comprised by multiple fragments of distinct gene products (see Supplementary Fig. S3). Consequently, annotations mapped from UniProtKB proteins can contain gaps, insertions or only a partial annotation region is covered in the structure. The 1D Protein Feature Viewer library provides different tools to manage such scenarios, including visualization of multiple sequences (see Supplementary Section S4).

## Funding

This work was supported by the National Science Foundation [DBI-1832184], the US Department of Energy [DE-SC0019749] and the National Cancer Institute, National Institute of Allergy and Infectious Diseases, and National Institute of General Medical Sciences of the National Institutes of Health under grant R01GM133198 (Principal Investigator: Stephen K. Burley).

*Conflict of Interest*: none declared.

## Supplementary Material

btaa1012_Supplementary_Data
