# Supplementary material for: RCSB Protein Data Bank 1D tools and services
Source: Bioinformatics. 2020 Dec 27;36(22-23):5526–7. doi: 10.1093/bioinformatics/btaa1012 (PMC8016458; doi:10.1093/bioinformatics/btaa1012)
Supplement: btaa1012_Supplementary_Data [file btaa1012_supplementary_data.docx]

**S1 Requesting alignments query and response format**

| Right-top code shows the GraphQL object used to request residue level mappings from the UniProtKB entry P01112 to NCBI RefSeq database. The main parameters for alignment requests are the origin database (**from**), the destination database (**to**) and the entry database Id for which alignments are being requested (**queryID**). The parameters identifying origin and destination databases have one of the next 4 values:   - **UNIPROT** (UniProtKB proteins) - **PDB_ENTITY** (PDB Entity sequences) - **PDB_INSTANCE** (PDB Instance chains) - **NCBI_PROTEIN** (NCBI RefSeq proteins) - **NCBI_GENOME** (NCBI Nucleotide chromosomes)   The parameter that defines the entry database to be mapped -**queryId**- needs to be a valid identifier in the origin database. The convention for identifying PDB entities is the concatenation of the PDB code and Entity Id with the delimiter “_” (*e.g.* 2UZI_3). To identify PDB instances, PDB codes are concatenates with chain label_asym_id using the delimiter “.” (*e.g.* 2UZI.C). It is important to highlight that the service does NOT use author_asym_id (a.k.a chain ID) but label_asym_id.  Right-bottom code shows the format in which alignments are encoded. Alignments are provided as an array of elements (**target_alignment**) that can be mapped to the requested entry (**queryId)**. The identifier in the destination database is encoded in the filed **target_id.** In this example, NP_789765, NP_001304983, NP_005334, and NP_001123914 are 4 NCBI RefSeq entries that can be mapped to the UniProtKB sequence P0111. The residue level mappings are encoded in an array (**aligned_regions)** of elements that describe the start and end position of the aligned residues in the query (origin) and target (destination) sequences. | \| Query: {  alignment(  from:***UNIPROT*** to:***NCBI_PROTEIN*** queryId:**"P01112"** ){  target_alignment {  target_id  aligned_regions {  query_begin  query_end  target_begin  target_end  }  }  } } \| \| --- \| \| Response: {  **"data"**: {  **"alignment"**: {  **"target_alignment"**: [  {  **"target_id"**: **"NP_789765"**,  **"aligned_regions"**: [  {  **"query_begin"**: 1,  **"query_end"**: 151,  **"target_begin"**: 1,  **"target_end"**: 151  }  ]  },  {  **"target_id"**: **"NP_001304983"**,  **"aligned_regions"**: [  {  **"query_begin"**: 149,  **"query_end"**: 189,  **"target_begin"**: 70,  **"target_end"**: 110  }  ]  },  {  **"target_id"**: **"NP_005334"**,  **"aligned_regions"**: [  {  **"query_begin"**: 1,  **"query_end"**: 189,  **"target_begin"**: 1,  **"target_end"**: 189  }  ]  },  {  **"target_id"**: **"NP_001123914"**,  **"aligned_regions"**: [  {  **"query_begin"**: 1,  **"query_end"**: 189,  **"target_begin"**: 1,  **"target_end"**: 189  }  ]  }  ]  }  } } \| |
| --- | --- | --- | --- |

**S2 Requesting residue level mappings from NCBI chromosomes to proteins**

This example describes how residue level mappings between protein sequences and genome nucleotides are encoded and how to assemble the final alignment from the delivered data. For that, we have requested the alignments for all PDB entity sequences that fall within Human chromosome 1 (NCBI Nucleotide code NC_000001) and analyze one of the fetched PDB entity targets (6I64_1), *i.e.* one of the elements of the **target_alignment** array (see Section S1).

| Response: {  **"orientation"**: -1,  **"target_id"**: **"6I64_1"**,  **"aligned_regions"**: [  {  **"query_begin"**: 216568024,  **"query_end"**: 216567988,  **"target_begin"**: 5,  **"target_end"**: 17,  **"exon_shift"**: [  216564380,  216564379  ]  },  {  **"query_begin"**: 216564378,  **"query_end"**: 216564219,  **"target_begin"**: 18,  **"target_end"**: 71,  **"exon_shift"**: [  216519421,  216519420  ]  },  {  **"query_begin"**: 216519419,  **"query_end"**: 216519152,  **"target_begin"**: 72,  **"target_end"**: 161,  **"exon_shift"**: [  216507183,  216507182  ]  },  {  **"query_begin"**: 216507181,  **"query_end"**: 216506942,  **"target_begin"**: 162,  **"target_end"**: 241,  **"exon_shift"**: []  }  ] } | The first aligned region begins at the PDB Entity 6I64_1 residue 5 and the Human Chromosome 1 (NCBI Id: NC_000001) nucleotide index 216568024 (yellow highlighted fields). Notice that the **orientation** value is -1 meaning that the PDB Entity maps with the negative DNA strand and nucleotide indexes will be aligned in descending order. Thus, residue 5 will be mapped to the [216568024, 216568023, 216568022] nucleotide indexes (notice the descending order). Following the 3 to one mapping between nucleotides and protein residues, in this example, the last nucleotide triad aligned with PDB Entity residue 17 (**target_end**) would surpass the **query_end** nucleotide index 216567988 by two positions. In those scenarios where the last nucleotide triad surpasses the end position of the aligned region by 1 or 2 positions, the missing nucleotide indexes will be available in the **exon_shift** array. Therefore, in this example, residue 17 will be aligned with [216567988, 216564380, 216564379] nucleotide indexes. It is worth to notice that, in this case, the **exon_shift** nucleotides (216564380, 216564379) are located just next to the beginning position of the next aligned region (chromosome nucleotide index 216564378). In fact, from a genome perspective they belong to the next exon of the transcript. In some cases the shifted nucleotides may comprise a whole exon themselves, *i.e.* an exon of 1 or two nucleotides and thus, this exon will not be encoded as an element of the **aligned_regions** array. In these situations, the number of aligned regions will not match with the number of gene exons. |
| --- | --- |

**S3 Entry mapping and sequence alignments between the different sequence databases**


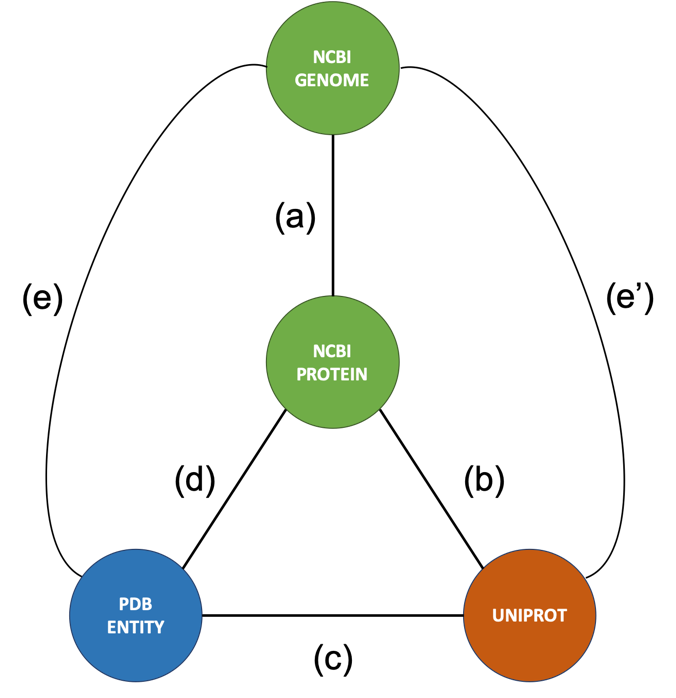


**Figure S1 Sequence alignments between databases**

1. NCBI RefSeq (Sharma, et al., 2019) provides mappings and alignment ranges between proteins, transcripts and their genome coordinates. All this information can be collected through the NCBI web services API.
2. Database entry mapping between NCBI RefSeq and UniProt proteins are collected from the Protein Information Resource (Wu, et al., 2003), a member of the UniProt consortium. Sequence alignment between mapped NCBI RefSeq and UniProt proteins are computed using the Smith-Waterman algorithm as implemented in BioJava (Lafita, et al., 2019) using the identity matrix as residue alignment weights, open gap penalty value 5 and extend gap penalty value 0.
3. Database entry mapping and sequence alignments between PDB Entity sequences and UniProt are collected from the Structure Integration with Function, Taxonomy and Sequence (SIFTS) resource (Dana, et al., 2019).
4. Database entry mapping between PDB Entities and NCBI RefSeq proteins is compiled combining (c) and (b). Then, residue level alignments are computed using the Smith-Waterman algorithm as implemented in BioJava using the identity matrix as residue alignment weights, open gap penalty value 5 and extend gap penalty value 0.
5. e’) Genome location of PDB Entities and UniProt proteins are mapped through their relationships with NCBI RefSeq proteins, *i.e.,* combining (d)+(a) and (b)+(a), respectively.

**S4 1D Protein Feature Viewer**

The main purpose of developing the 1D Protein Sequence Viewer was to provide a library that could optimally display annotations coming from multiple protein references when one-to-many relationships between database entries and partial alignments were present. These are common situations when mapping PDB structures to UniProtKB or NCBI sequences. In many cases, experimentally-determined structures do not cover the entire native protein sequence, and thus, some positional annotation might be cut when mapped to PDB structures. Another commonly occurring case, are structures that for crystallographic or other reasons a sequence insertion was introduced and thus, it might be composed by multiple protein sequences. In these cases, positional annotations might be affected by insertions and/or deletions and can be mapped from multiple references. Figure S1 shows how the 1D Protein Feature Viewer can represent insertions or incomplete annotations as dashed lines (insertions) or white circles (missing region), respectively.


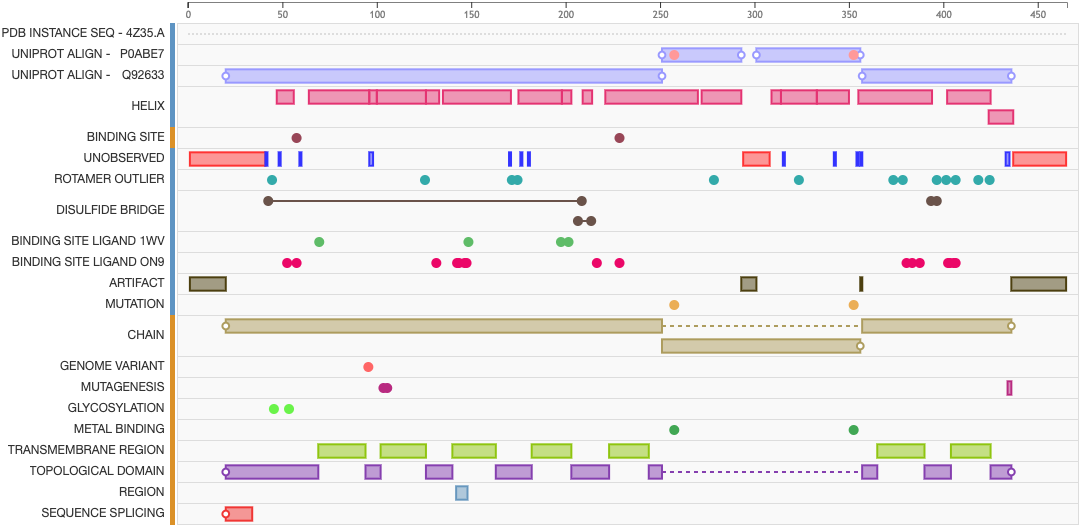


**Figure S2. 1D Protein Feature Viewer.** Visualization of biological and structural features for the Human Lysophosphatidic Acid Receptor (PDB code: 4z35 chain A). Dashed lines between blocks indicates that in the original reference the local annotation is connected and thus, an insertion occurs when mapped to the PDB structure. white circles over the start (or end) of a block indicates that a feature region in its original reference is missing and thus, it does not start (or end) in that position but before (or after).

In addition, the 1D Protein Feature Viewer allows display of multiple sequence alignments to increase the coverage of a reference sequence with respect to a particular database. For example, in Figure S3 two UniProt sequence alignments (UniProt accessions: P0ABE7 and Q92633) are displayed to increase the coverage of the PDB Entity 4Z35_1 with respect to UniProt sequences. To the best of our knowledge, displaying multiple sequence alignments is not available in other tools that display positional annotations.


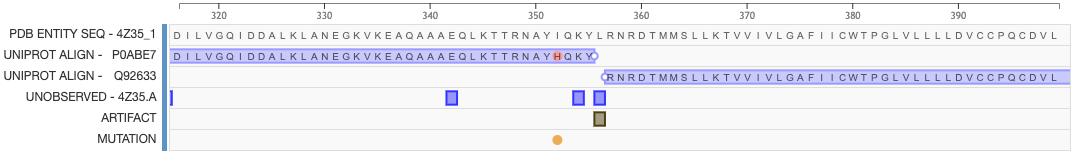


**Figure S3. Displaying Multiple Sequences.** The PDB structure 4z35 is mapped to the UniProtKB entry Q92633 however, it contains and insertion from the UniPrtoKB entry P0ABE7 (Soluble cytochrome b562, E. Coli protein).

**References**

Dana, J.M.*, et al.* SIFTS: updated Structure Integration with Function, Taxonomy and Sequences resource allows 40-fold increase in coverage of structure-based annotations for proteins. *Nucleic Acids Res* 2019;47(D1):D482-D489.

Lafita, A.*, et al.* BioJava 5: A community driven open-source bioinformatics library. *PLoS Comput Biol* 2019;15(2):e1006791.

Sharma, S.*, et al.* The NCBI BioCollections Database. *Database (Oxford)* 2019;2019.

Wu, C.H.*, et al.* The Protein Information Resource. *Nucleic Acids Res* 2003;31(1):345-347.
